# Cold Plasma Generates a Localized Inflammatory Response and Promotes Muscle Repair

**DOI:** 10.1002/adtp.202500097

**Published:** 2025-05-20

**Authors:** Carly J. Smith, Amanda R. Watkins, Abigail A. Lucas, Arianna J. Moniodes, Conn Ritchie, Thomas P. Thompson, Thomas P. Schaer, Brendan F. Gilmore, Noreen J. Hickok, Theresa A. Freeman

**Affiliations:** Department of Orthopedic Surgery Research, Thomas Jefferson University, 1015 Walnut Street Suite 501, Philadelphia, PA 19107, USA; New Bolton Center Department of Clinical Studies, University of Pennsylvania School of Veterinary Medicine, 382 West Street Road, Kennett Square, PA 19348, USA; Department of Orthopedic Surgery Research, Thomas Jefferson University, 1015 Walnut Street Suite 501, Philadelphia, PA 19107, USA; Department of Orthopedic Surgery Research, Thomas Jefferson University, 1015 Walnut Street Suite 501, Philadelphia, PA 19107, USA; School of Pharmacy, Queen’s University Belfast, Belfast BT9 7BL, UK; School of Pharmacy, Queen’s University Belfast, Belfast BT9 7BL, UK; New Bolton Center Department of Clinical Studies, University of Pennsylvania School of Veterinary Medicine, 382 West Street Road, Kennett Square, PA 19348, USA; School of Pharmacy, Queen’s University Belfast, Belfast BT9 7BL, UK; Department of Orthopedic Surgery Research, Thomas Jefferson University, 1015 Walnut Street Suite 501, Philadelphia, PA 19107, USA; Department of Orthopedic Surgery Research, Thomas Jefferson University, 1015 Walnut Street Suite 501, Philadelphia, PA 19107, USA

**Keywords:** cold plasma, muscles, orthopedic surgery, reactive species, tissue regeneration

## Abstract

The FDA-approved Renuvion cold plasma device is currently used for dermal skin tightening procedures and subdermal tightening after liposuction. Anecdotally, patients report improved tissue healing outcomes following treatment. The most likely explanation for this is plasma-generated reactive species which are inflammatory but also activate cellular signaling pathways, stimulate antioxidant responses, and activate immune cells. In this study, we aimed to determine the immediate and long-term molecular effects of a single plasma treatment on surgically injured muscle and the soft tissue envelope. We used RNA sequencing, histology, and immunohistochemistry to determine changes to the tissue following treatment. Neutrophils and mast cells rapidly mobilize 6 h after treatment in conjunction with an upregulated cellular antioxidant response. Additionally, genes identified by RNAseq indicate upregulated pro-regenerative muscle-tissue-protective gene transcripts and downregulated apoptotic pathway transcripts in the muscle tissue 6 h after treatment. The histology and RNAseq results from 4- and 14-days post plasma treatment indicate that these early inflammatory and antioxidant events drive muscle regeneration to skew toward myogenic differentiation over adipogenesis. Thus, we conclude that a single plasma treatment results in an immediate inflammatory and antioxidant response that enhances long-term muscle fiber repair through reduced adipogenesis.

## 1. Introduction

Tissue healing following surgical procedures can pose several challenges depending on patient demographics, comorbidities, anatomical location, extent of damage, presence of infection, and a host of other considerations. Enhancing the ability of tissues to heal after surgery would provide better patient outcomes and could prevent additional medical complications and costs.^[1]^ The relatively new technology of cold atmospheric plasma may help achieve these outcomes. To date, cold atmospheric plasma devices have been tested for a variety of medical applications including wound healing, cancer treatment, and infection mitigation.^[2,3]^ Cold plasma is an ionized gas generated when a high energy electric field is applied to an inert gas, nitrogen or air. This produces a cocktail of reactive products including reactive oxygen and nitrogen species (ROS/RNS), ions, electrons, heat, UV radiation, and electromagnetic disturbances.^[4–6]^ Of these products, ROS and RNS are the major components that drive plasma bioactivity.^[4,7,8]^ It has been shown that the bioactivity of plasma is mitigated when a reactive species scavenger is used to reduce the effect of these species.^[9,10]^ Clinical and non-clinical studies using cold plasma have demonstrated that tissue responses vary depending on the type of plasma used, the treatment time (dose), and the tissue type.^[11,12]^ Based on these factors, a range of plasma-generated outcomes including cancer and bacterial cell killing, enhancement of angiogenesis, cell infiltration, and wound healing have been demonstrated both in vitro and in vivo.^[11,13–17]^ Mechanistic investigations of plasma bioactivity have revealed that plasma-generated ROS/RNS can also activate a DNA damage response, antioxidant response, and anti-inflammatory response while also promoting cell proliferation, survival, and differentiation in eukaryotic cells.^[18–23]^

While these studies highlight the potential of cold plasma in medical applications, the scientific literature detailing these results consists of a compendium of studies encompassing thousands of different devices operating in an infinite number of settings. Unfortunately, this confounds the ability to generate repeatable, translational outcomes in a clinical setting. There are however a few plasma devices that have been characterized for commercialized use to operate in a reproducible manner, such as the kINPen (a class IIa medical device approved for use in Europe), which is reported to have significant wound-healing capabilities.^[14]^ However, in the United States, only the Renuvion plasma device (formerly Bovie J-Plasma) is FDA-approved. Its approved uses include: 1) general use of cutting, coagulation, and ablation of soft tissue, 2) dermal resurfacing procedures for the treatment of severe wrinkles and rhytids, 3) improvement of the appearance of lax skin in the neck and submental regions, and 4) coagulation of subcutaneous soft tissues following liposuction for aesthetic body contouring.^[14,24–29]^ In addition to these FDA-stated indications, clinicians have used and anecdotally reported improved success in grafting procedures, breast reductions, and skin suppleness while using the device.^[3,30,31]^ Further, Renuvion has been shown to reduce skin injury depth and enhance granulation tissue deposition, which suggests enhanced tissue regeneration.^[7,32]^ These reported findings suggest the activation of reactive species-driven pathways that stimulate these pro-regenerative outcomes.^[14,24,25]^ However, an in-depth analysis of how the Renuvion device mechanistically drives tissue repair and wound healing processes in vivo has not been performed.

Generally, surgical procedures cut through not just the skin, but into the soft tissue envelope consisting of subcutaneous tissue, fascia, nerves, blood vessels, and muscle causing tissue damage as a result. Muscle healing after injury, including surgical incision, is a well-defined and highly regulated process of degeneration, inflammation, regeneration, and repair.^[33,34]^ Injury drives immune cell invasion and inflammatory cytokine production, which are critical for initiating the regeneration process.^[35]^ More specifically, these inflammatory stimuli lead to the activation of muscle stem cells (satellite cells), which begin rapidly dividing and differentiating to repair the damaged tissue. Importantly, the rapid resolution of inflammation is required to ensure satellite cells direct the restoration of functional muscle (myogenesis) rather than generating fibrotic or fatty replacement tissue (adipogenesis) during healing.^[36–43]^ Treatment of muscle tissue by cold plasma-generated reactive species stimulate an transient inflammatory burst – similar to that produced by activation of immune cells after injury – but in a more intense and rapid manner. These species can also trigger surrounding cells to produce a robust antioxidant response as a protective mechanism.^[20,21]^ This oxidative burst enhances and speeds ROS-induced signaling which stimulates angiogenesis and an immune response including neutrophil and macrophage activation, exceeding that which is produced by the body alone.^[5,14,40,41]^ This enhanced immune response to plasma treatment can generate an opportunity for more efficient satellite cell activation and thus more effective muscle repair after surgical injury.

Based on this premise, we chose to investigate the molecular effects of single plasma treatment on surgically injured muscle and assess how this would alter the repair and healing response. We utilized a rat model of muscle injury by making an open surgical approach to the mid-diaphysis of the femur and assessed the molecular (RNA sequencing) and pathologic (histology, immunohistochemistry) responses of injured muscle tissue after a single treatment of Renuvion cold plasma and compared them to untreated muscle. We assessed pathway and gene activation 6 h after treatment to determine the initial molecular responses to plasma. To determine the effect on muscle healing at longer time points, we used a more challenging revision surgery model and collected tissue 4- and 14-days after a secondary revision surgery. The results from this study indicate that a single treatment of cold plasma from the Renuvion device can influence the initial inflammatory response and trigger tissue-protective gene expression. The use of cold plasma on muscle after surgical incision induced muscle injury resulted in a decrease in adipogenesis gene transcription and a decrease in fatty deposition in the repairing muscle.

## 2. Results

### 2.1. Cold Plasma Treatment of the Soft Tissue Envelope Results in Iron Oxidation and Collagen Fibril Disruption

**Figure 1.**
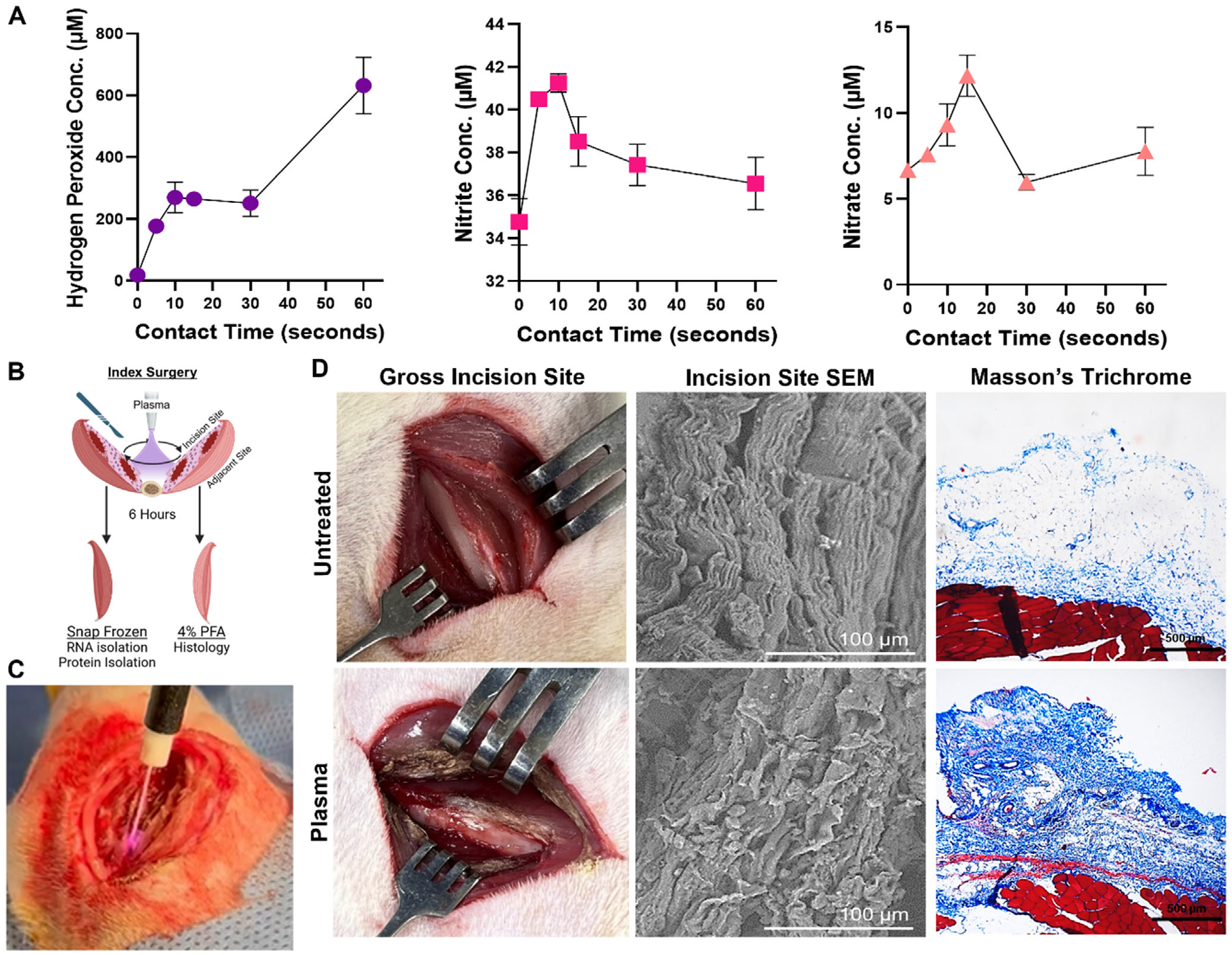
Cold plasma treatment of the soft tissue envelope results in iron oxidation and collagen fibril disruption. (A) Hydrogen peroxide, nitrite, and nitrate concentrations (μM) in plasma-treated deionized water. Error bars represent ±SD, *n* = 3 wells/timepoint. (B) Diagram of treatment and tissue collection protocol. (C) Image of cold plasma treatment of incision site tissue. (D) Representative gross images of the untreated and plasma-treated incision site. Scanning electron microscope (SEM) images of the untreated and plasma-treated incision site connective tissue immediately following surgery. High-magnification images of the untreated and plasma-treated incision site tissue, 6 h post-surgery. Stained with Masson’s Trichrome.

The Renuvion device is a commercially available plasma generation device that includes both an electrosurgical generator and an interchangeable handpiece. For consistency, all in vitro and in vivo experiments were conducted using the same parameters: 28 W power and 4 L min^−1^ helium flow rate. We found that when water is treated with the Renuvion cold plasma device, while some nitrites and nitrates are generated, hydrogen peroxide is the predominant type of reactive species produced by the plasma (Figure 1A). To investigate the effect of plasma treatment on tissues within the surgical site, we employed a rat surgical incision model. We generated an open surgical approach to the femur, and the tissue was either untreated or treated with plasma. All incisions regardless of treatment received a saline rinse. After the rinse, the incisions were closed and 6 h later the tissues were collected for RNA isolation or histology as depicted in Figure 1B. An example of how the plasma stream looks when it is discharged on muscle tissue within an incision is depicted in Figure 1C. Immediately following plasma treatment the red, blood-rich muscle tissue was visibly oxidized, changing to a brown color, likely due to the oxidation of iron in heme (Figure 1D). Visualization of untreated muscle tissue by scanning electron microscopy (SEM) revealed parallel bundles of highly structured fibrils organized in wave patterns (Figure 1D). Fibrils treated with plasma appeared to lose their organized structure and exhibited ruffling and condensation (Figure 1D). Interestingly, in the Masson’s trichrome stained muscle sections, while the depth of acidification (blue stain) of the tissue at the incision was similar between untreated and plasma-treated groups (Figure S1A), the plasma-treated tissue stained a darker blue (Figure 1D). These results suggest that the plasma tissue interaction is comprised of both the plasma-generated ROS penetration that causes tissue acidification, and the condensation of the connective tissue fibril structure that is observed through SEM.

### 2.2. Plasma Recruits Innate Inflammatory Cells to the Treated Site

**Figure 2.**
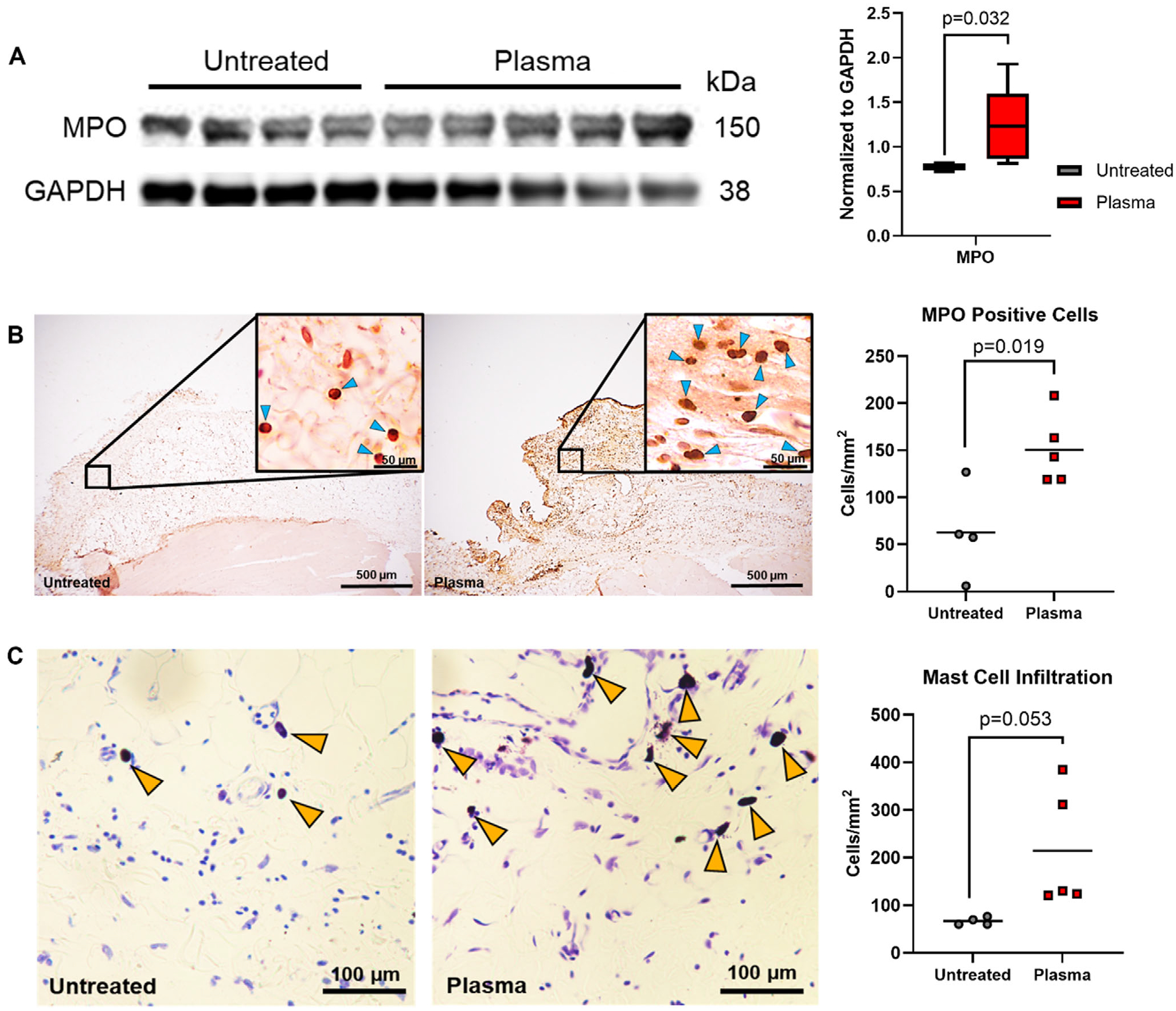
Plasma recruits innate inflammatory cells to the treated site. (A) Immunoblot of myeloperoxidase (MPO) protein expression 6 h after surgery in untreated (*n* = 4) and plasma-treated (*n* = 5) incision site muscle. Quantification of relative protein expression of MPO in untreated and plasma-treated incision site muscle. Relative expression compared to GAPDH. P-values determined using Mann Whitney test (*p* < 0.05). Data are presented as min. to max. (B) Representative images of sectioned muscle at the incision site of untreated and plasma-treated animals stained using immunohistochemistry and a myeloperoxidase (MPO) antibody (1:500) to identify neutrophils. Insets show MPO+ neutrophils (dark brown) marked by light-blue arrows. Non-MPO+ cells are counterstained with hematoxylin and appear light brown/purple. Quantification of IHC average MPO+ cells mm^−2^ in the incision site of untreated (*n* = 4) and plasma-treated (*n* = 5) muscle tissue. P-values determined using the student’s t-test (*p* < 0.05). (C) Representative images of muscle stained with toluidine blue to identify mast cells in untreated and plasma-treated incision site fibrotic regions. Mast cells are marked with yellow arrows. Quantification of average mast cells mm^−2^ in toluidine blue-stained untreated (*n* = 4) and plasma-treated (*n* = 5) fibrotic regions of incision sites. *n* = 5 images/animal. P-values determined using student’s t-test (*p* < 0.05).

The muscle response to injury is characterized by three major phases following the initial injury stage: inflammation, regeneration, and repair.^[44]^ The inflammation stage lasts from 2–24 h post-injury and is characterized by an influx of neutrophils which secrete pro-inflammatory cytokines, proteases, and free radicals that further recruit macrophages to remove debris and activate satellite cells.^[44]^ Thus, we hypothesized that there would be increased neutrophils at the injured site 6 h after injury. Interestingly, in the absence of injury, plasma is also known to activate and recruit neutrophils.^[40]^ We found that 6 h after plasma treatment myeloperoxidase (MPO; a marker of neutrophils) protein levels were significantly increased (*p* = 0.032) when compared to untreated muscle, as measured by Western blot (Figure 2A). Immunohistochemistry confirmed that the number of neutrophils (light blue arrows) at the incision site was significantly increased (*p* < 0.001) with plasma treatment, whereas there was no significant increase in neutrophils at the incision site of untreated animals (Figure 2B).

To further elucidate the inflammatory effects of plasma treatment, we used toluidine blue staining to identify tissue-resident mast cells (yellow arrows) in the incision site. Plasma treatment induced a significant increase in mast cells in the treated area (Figure 2C). Tissue-resident mast cells, while often associated with allergic reactions, are also responsible for sensing damage and infection, and have recently been identified as a required cell type for initiating muscle regeneration after injury.^[42]^ Taken together, these results suggest that plasma treatment initiates an enhanced, but highly localized innate immune response within the surgical incision 6 h after treatment and that the responding cell types may help prime the muscle for regeneration after surgical injury.

### 2.3. Plasma Treatment Evokes a Tissue-Protective Response in Muscle Tissue

**Figure 3.**
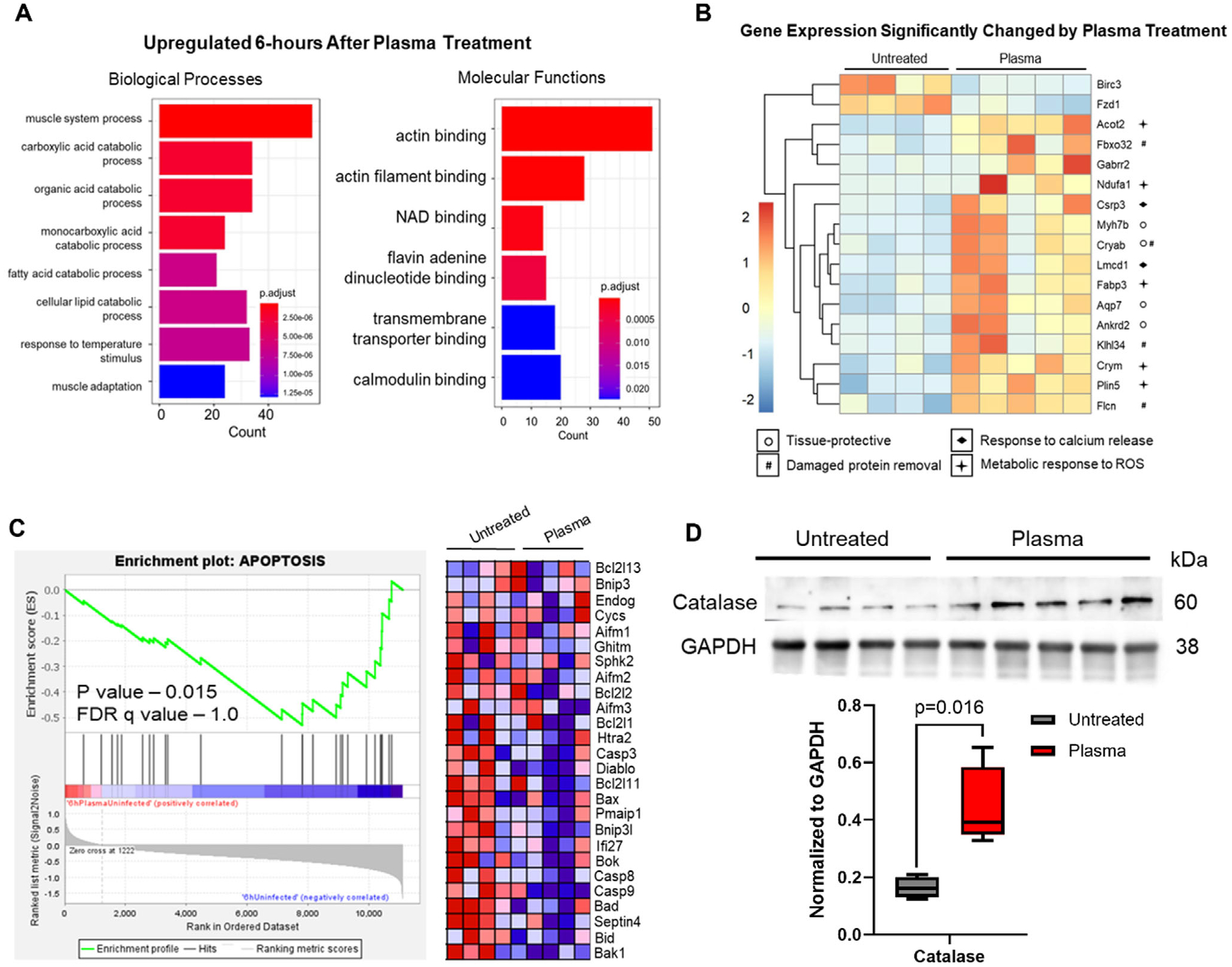
Plasma treatment evokes a tissue-protective response in muscle tissue. (A) Gene Ontology (GO) pathways from bulk RNA sequencing of incision site muscle that are significantly (*p* < 0.05) upregulated 6 h after plasma treatment. (B) Significantly up- and down-regulated gene transcripts 6 h after plasma treatment. Functional groups are defined by their respective symbols. Data presented as Log2Fold Change. (C) Gene Set Enrichment Analysis (GSEA) comparing plasma-treated incision site muscle vs untreated incision site muscle 6 h after surgery. MitoCarta curated gene list: APOPTOSIS. (D) Immunoblot of catalase protein expression 6 h after surgery in untreated and plasma-treated incision site muscle. Quantification of relative protein expression of catalase in untreated and plasma-treated incision site muscle tissue. Relative expression is compared to GAPDH and p-values were determined using Mann Whitney test (*p* < 0.05). Data are presented as Min. to Max. For all groups, *n* = 4 untreated and *n* = 5 plasma-treated rats.

While an appropriate inflammatory response is critical for muscle healing after injury, the tissue response to oxidative stress can either be detrimental or protective depending on the magnitude of the stress.^[43,45,46]^ We used bulk RNA sequencing of untreated and plasma-treated muscle to determine whether the tissue response to plasma was protective or detrimental. Gene Ontology (GO) enrichment analysis highlighted functional pathways and processes that were upregulated with plasma. The upregulated biological processes primarily included metabolic changes, including multiple catabolic processes similar to those associated with exercise (Figure 3A).^[47]^ In support of the relationship to exercise-like molecular changes, we also observed the upregulation of actin and NAD binding as well as calmodulin activity (Figure 3A). These pathways regulate muscle fiber adaptation and oxidative capacity in response to exercise through calcium-dependent transcriptional activation.^[43,47]^ Taken together, all the significantly modified gene transcripts trended toward positively influencing muscle healing (Figure 3B). To illustrate this, we used gene function to group the significantly changed transcripts as follows: tissue-protective genes (○), genes with roles in damaged protein removal (#), genes that respond to calcium release (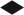), and genes that metabolically respond to ROS (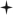) (Figure 3B).

The tissue-protective genes group includes: *Cryab*, an apoptosis inhibitor, *Ankrd2* an inhibitor of NF*κ*B signaling, and *Aqp7* a protector against ischemia.^[48–50]^ In addition, *Gabrr2* indicates a neuroactive ligand-receptor interaction that may represent increased neuronal, stromal, and T-regulatory cell crosstalk which is implicated in enhancing muscle repair (Figure 3B).^[51]^ The gene group with roles in damaged protein removal includes: *Klhl34*, which targets protein for degradation, *Fboxo32*, which clears damaged lysosomes, *Flcn*, which is important in lysosome activity, and *Cryab*, which binds misfolded proteins and inhibits apoptosis (Figure 3B).^[48,52–54]^ The third functional group includes genes that respond to calcium release. *Lmcd*, a calcineurin activator is upregulated. Calcineurin is activated by muscle damage-induced calcium release to coordinate the repair response.^[55]^ In coordination, *Csrp3*, a transcription factor that maintains muscle structure, is also upregulated.^[56]^ (Figure 3B). Finally, the fourth functional group includes genes that metabolically respond to ROS. Several metabolic response transcripts are upregulated in response to plasma including *Ndufa1* which promotes ubiquinone production, and *Crym* which promotes a shift from glycolysis to catabolism of fatty acids by *β*-oxidation.^[57–59]^ (Figure 3B). Additionally, several transcripts involved in fatty acid metabolism are upregulated in response to plasma including *Acot*2, which aids in free fatty acid production, *Fabp3*, a fatty acid binding protein that acts as an indicator of ER stress via the PERK-eIF2*α* pathway, and *Plin5* which induces FGF21, a longevity indicator.^[58,60,61]^ (Figure 3B). Only two genes were significantly upregulated in untreated muscle: *Birc3* and *Fzd1* (Figure 3B). Increased expression of *Fzd1* in aged mice inhibits myogenic differentiation of satellite cells.^[62]^ Increased expression of *Birc3* has been implicated in the protection of nerves after crush injury by inhibition of apoptosis, but interestingly it has no protective effect in response to reactive species-induced apoptosis.^[63]^ These findings, along with the GO functional changes in Figure 3A, suggest that plasma treatment triggers calcium signaling through calcineurin, alterations in mitochondrial metabolic activity, and muscle repair pathways above the levels that occur in normal muscle tissue when healing from surgery.

To more conclusively decipher how several genes work together to affect tissue function, we performed Gene Set Enrichment Analysis (GSEA). In support of the significant individual gene transcript changes in the treated tissue, GSEA revealed that the only significant pathway change in response to plasma treatment was the downregulation of apoptosis (Figure 3C). Based on these results, and the established protective antioxidant response known to be triggered by plasma-generated ROS, we performed a Western blot for the most stable tissue-associated antioxidant, catalase, and found that it is significantly (*p* = 0.016) upregulated with plasma treatment (Figure 3D). Upregulation of tissue-protective gene transcripts strongly suggests that the early (6 h) tissue response to plasma treatment is the creation of a pro-regenerative environment that may lead to improved muscle regeneration at later timepoints. We hypothesize that this effect is due to the tissue response to the local inflammatory signaling cascade caused by plasma treatment coupled with the upregulation of the antioxidant catalase. However, other factors may be involved.

### 2.4. Plasma Treatment Reduces Transcription of Inflammation and Adipogenesis-Associated Genes

**Figure 4.**
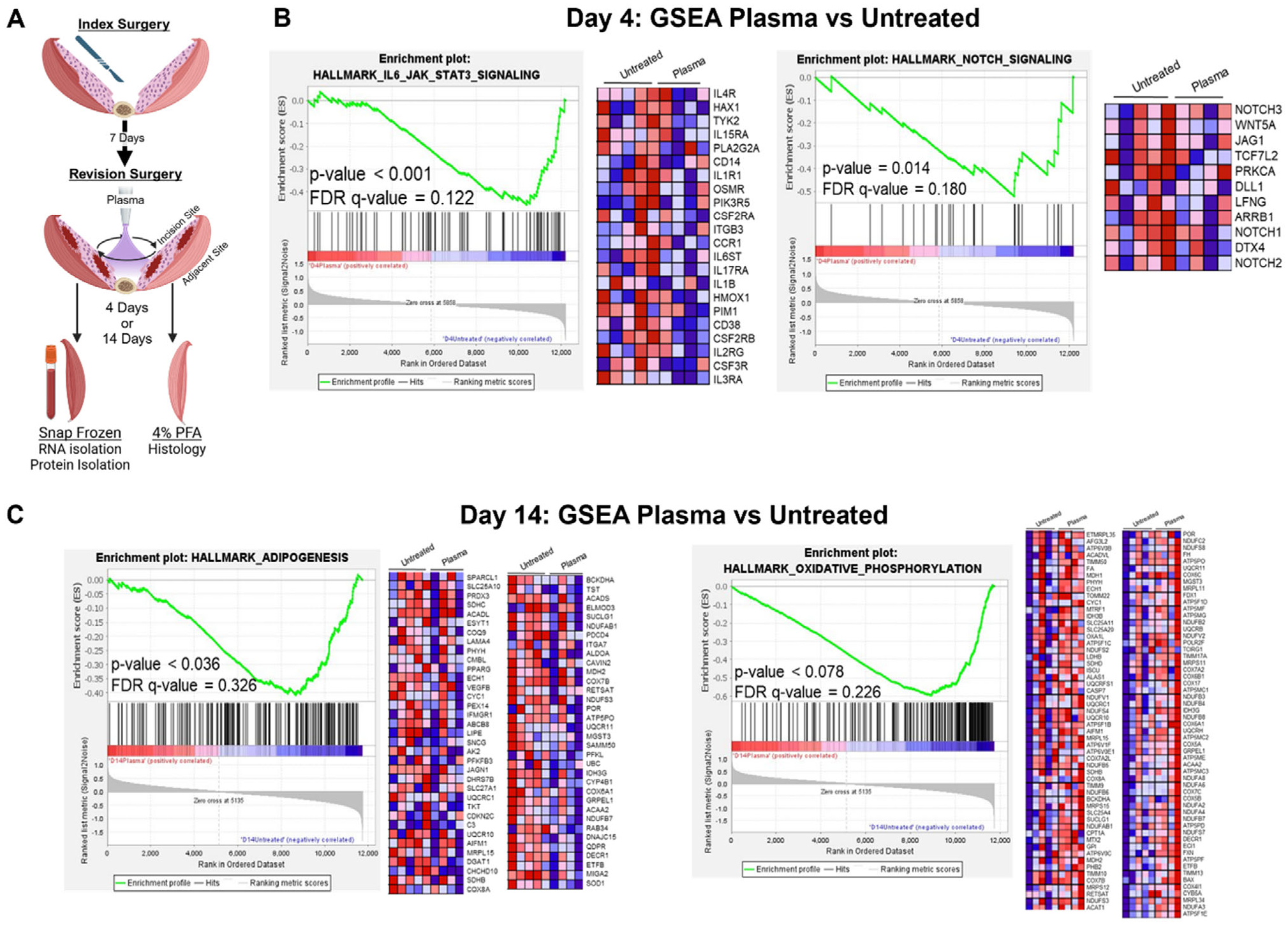
Plasma treatment reduces the transcription of inflammation and adipogenesis-associated genes. (A) Diagram indicating treatment and tissue collection protocol for rat revision model. (B) Gene Set Enrichment Analysis (GSEA) of bulk RNA sequencing results comparing plasma-treated incision site muscle to untreated incision site muscle 4 days after revision surgery. Hallmarks curated gene lists from MSigDB: HALL-MARK_IL6_JAK_STAT3_SIGNALING and HALLMARK_NOTCH_SIGNALING. (C) GSEA comparing plasma-treated incision site muscle to untreated incision site muscle 14 days after revision surgery. Hallmarks curated gene lists from MSigDB: ADIPOGENESIS and OXIDATIVE_PHOSPHORYLATION. For all groups, *n* = 5 untreated and *n* = 4 plasma-treated rats.

To test if stimulation of these molecular pathways by a single plasma treatment would have a more lasting effect on the outcome of muscle healing, we employed a more challenging surgical model. This second model has two separate surgical procedures: an index surgery during which all rats receive an identical operation, and a revision surgery 1 week later (Figure 4A). The revision surgery reopens the original incision site and either no treatment (control) or plasma treatment is administered. Tissue is collected 4- or 14-days post-revision for analysis. Clinically, revision surgery is more damaging to tissue and associated with increased risk for complications including poor or delayed wound healing and infection, making this model more suitable to determine if plasma treatment enhances muscle healing.^[64–66]^

We used bulk RNA sequencing of untreated and plasma-treated muscle to determine transcriptional changes in the muscle 4- and 14-days post-revision. The GSEA results from 4 days post-revision revealed a significant downregulation in IL-6 JAK/STAT and Notch signaling (Figure 4B). The activation of the IL-6 JAK/STAT pathway is necessary for muscle repair, however, prolonged activation is often associated with increased inflammation and adipogenesis in muscle tissue.^[67]^ Similarly, the Notch pathway is important for satellite cell activation in the short term, but if present for too long can suppress myogenesis by preventing satellite cells from differentiating.^[68]^ In coordination with these results, cytokine levels of IL-4 protein, a potent activator of the JAK/STAT signaling pathway, were also significantly decreased 4-days post-revision surgery in plasma-treated rats (Table 1).^[69]^

The GSEA results from 14 days post-treatment show significant downregulation of both adipogenesis and oxidative phosphorylation signaling pathways (Figure 4C). Oxidative phosphorylation of immune cells represents increased inflammatory status, while in muscle decreased oxidative phosphorylation is often associated with slow-muscle fiber generation.^[39,70]^ In support of reduced inflammation associated with plasma treatment, cytokine expression levels of CINC-2, IL-13, L-Selectin, and RAGE, which are largely involved in inflammatory and leukocyte homing responses, were also significantly downregulated (Table 1).^[69,71,72]^ These findings suggest that plasma treatment has long-term healing effects on muscle tissue that may drive quicker resolution of inflammation to speed the muscle regenerative process toward functional myogenesis and away from adipogenesis.

### 2.5. Plasma Treatment Decreases Fat Accumulation and Promotes Healthy Muscle Regeneration

**Figure 5.**
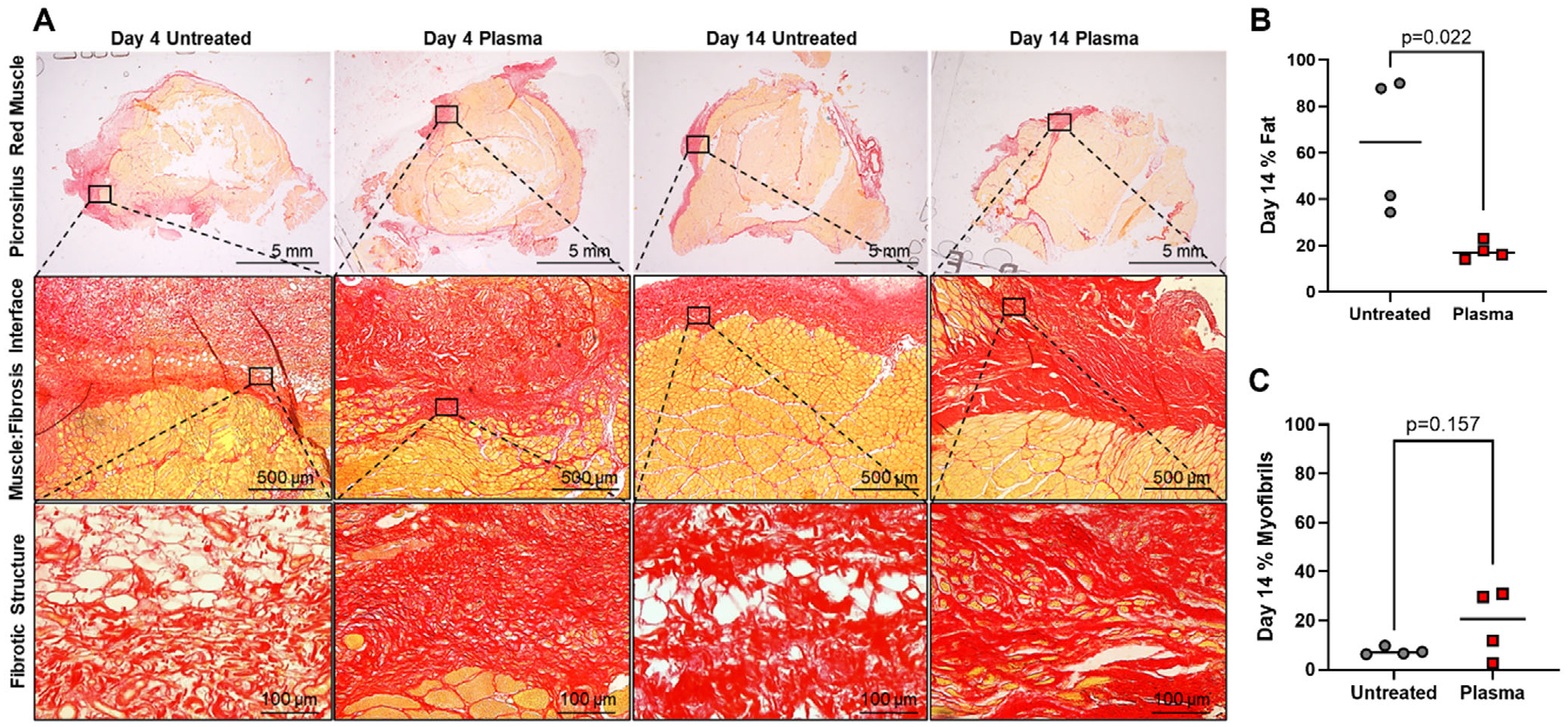
Plasma treatment reduces the long-term accumulation of fat in healing connective tissue and promotes healthy muscle regeneration. (A) Representative images of Picrosirius-red stained incision site muscle tissue in untreated and plasma-treated animals. Collagen fibers stain red. Muscle fibers stain yellow. The Muscle: Fibrosis Interface represents the location at the incision site where the muscle fibers contact the collagen layer. Purple arrows mark areas where the collagen fibers integrate between muscle fiber bundles. The asterisk (*) marks where there are identifiable inter-fibrotic fat deposits. High-magnification Fibrotic Structure images show detail in the structure of incision site cartilage fibers and inter-fibrotic fat (marked with an asterisk). Inter-fibrotic muscle fibers are stained yellow. (B) Quantification of percent fat within the fibrotic tissue in untreated and plasma-treated rats at day 14 post-revision surgery. P-values determined using the student’s t-test (*p* < 0.05). (C) Quantification of percent myofibrils within the fibrotic tissue in untreated and plasma-treated rats at day 14 post-revision surgery. P-values determined using the student’s t-test (*p* < 0.05).

Muscle repair can result in myogenesis, fibrogenesis, or adipogenesis depending on the efficiency of repair.^[36–39]^ Based on the RNA sequencing results, we used picrosirius red staining to investigate the repair of the incision site muscle using histology (Figure 5A). This method specifically stains collagen connective tissue (red) and healthy muscle fibers (yellow). We stained a cross–section of the vastus lateralis muscle, and the staining exhibited a band of red collagen tissue at the incision site, which represents the area of ongoing muscle repair (Figure 5A). There was no significant difference in the size of this region between the untreated and plasma-treated groups (Figure S1B). Higher magnification images of this region (black box) show that the differences in collagen compaction observed in the 6 h samples (Figure 1D) are maintained at these longer time points. In this region of active tissue repair, different cell/tissue types are visible as: white (fat), yellow (muscle), and red (fibrotic). Thus, to assess the outcome of repair, we quantified the percent fat and determined that at day 14 (Figure 5B), but not at day 4 (Figure S1C) the untreated animals had significantly more inter-fibrotic fat present in the repairing area. These results suggest that the single plasma treatment at surgical revision has a long-term impact that promotes better muscle repair. We also quantified the percent myofibrils in the healing region, and while there was no significant difference at either day 4 (Figure S1D) or day 14 (Figure 5C), there is a trending increase in myofibrils in the region, which we hypothesize would become significantly different with plasma treatment at longer time points. These findings further support the conclusion that plasma treatment is beneficial and has long-term effects, likely mediated through a rapid increase in inflammatory cytokine signaling, that is quickly quenched by antioxidant production which produces enhanced cell protection and reduced cell death. This in turn promotes enhanced stem cell differentiation into muscle to promote better outcomes in muscle regeneration following injury.

## 3. Conclusion

Here we show that cold plasma treatment of the surgical soft tissue envelope stimulates an increased inflammatory reaction accompanied by tissue protective signaling. Together these signaling events drive muscle repair toward myofibril regeneration over adipogenesis. The direct interaction of the plasma stream with the loose connective tissue surrounding the muscle results in collagen fibril condensation and bunching. After 6 h there is a significant influx of innate immune cells, such as neutrophils and tissue-resident mast cells, localized to the plasma treatment site. Molecularly, these events are accompanied by significant upregulation of tissue-protective antioxidants, decreased apoptosis, and enhanced fatty acid metabolism. Evidence that this single treatment with cold plasma leads to an enhanced regenerative response is confirmed when we employ the more challenging surgical revision model. The long-term outcomes of cold plasma treatment in this model reveal decreased adipogenesis gene enrichment and less adipose tissue within the regenerating muscle. Taken together this study serves to identify the molecular events initiated by a single cold plasma treatment to enhance muscle tissue regeneration over adipogenesis. It may also represent an interesting explanation for the positive clinical outcomes associated with Renuvion and other cold plasma devices when promoting tissue healing.

Previously, several different gas plasma devices have been shown to promote enhanced wound healing when applied to different tissues.^[14–17]^ However, the only FDA-approved plasma device for use on humans, the Renuvion device, has not been characterized for wound-healing effects, despite anecdotal accounts from patients who report enhanced healing following fat graft procedures.^[3,7,30–32]^ In this study, we utilized a rat model of surgical incision to determine the immediate, short-term, and long-term effects of Renuvion plasma treatment on muscle repair. The physical interaction of plasma with the treated tissue, characterized by SEM and histology, was consistent with local oxidation and the efficient transfer of heat.^[26]^ This finding is demonstrated by the tissue discoloration (brownish coloration consistent with iron oxidation) and dehydration visible during surgery, resulting in observable compaction and condensation of the collagen fibrils. Both patient accounts and the established mechanism of action for the Renuvion device describe skin “tightening” consistent with this effect.^[26,31]^ In addition to these immediate effects, we and others report that plasma devices, including the Renuvion device, produce highly bio-active reactive species including both reactive oxygen (ROS) and reactive nitrogen (RNS) species.^[4–7]^ The high-voltage plasma energy causes additional species generation as it interacts with molecules specific to each target surface. For example, proteins can be nitrosylated, lipids peroxidized, and inorganic molecules (Na, Cl, etc) can be combined with oxygen or nitrogen ions.^[73–75]^ This combination of effects differentiates plasma treatment from the simple addition of a bolus of hydrogen peroxide into a wound. The resultant cell signaling from plasma can drive cell proliferation, differentiation, and tissue repair pathways that trigger tissue healing.^[76,77]^

While surgical techniques try to avoid muscle trauma by using blunt dissection along natural fascial planes, an injury response is unavoidable. In fact, the innate immune response is essential to wound healing, as leukocyte populations are responsible for secreting the signals for blood clotting, scar formation, tissue regeneration, and angiogenesis.^[78]^ Our study suggests reactive species locally generated by Renuvion plasma treatment increase the innate immune inflammatory response and simultaneously generate a tissue-protective antioxidant response. Together, this drives muscle regeneration.^[40–42]^

Muscle is a naturally regenerative tissue that strengthens through daily repeated cycles of injury and repair, such as exercise.^[46,79,80]^ Molecularly during these cycles, calcium signaling is rapidly initiated by injury to recruit muscle-resident satellite cells that begin the process of muscle regeneration.^[46,79,80]^ Interestingly, our analysis of significantly upregulated gene transcripts 6 h after plasma treatment identifies many genes that are involved in these same processes including calcium release, muscle repair, and antioxidant response pathways. As reactive species signaling is tightly intertwined with calcium signaling, an enhanced calcium release following plasma treatment would not be unexpected.^[81]^ Indeed, in this context, the enhanced mobilization of repair pathways observed at the 6 h timepoint seems to indicate that the response to plasma treatment may operate through these natural injury and repair pathways.

Based on previous work on muscle regeneration, we selected our timepoints to reflect the three main stages of injury and repair: inflammation, regeneration, and remodeling.^[79]^ The inflammation stage, which dominates the first 48 h after injury, is characterized in our model by the significant influx and expansion of innate immune cell populations in the incision site, particularly with plasma treatment. The regeneration stage, which occurs from 2–10 days post-injury, is characterized by the activation of either satellite cells or fibro-adipogenic progenitors (FAPs) which can differentiate into pro-myogenic myofibroblasts or adipocytes.^[82]^ Finally, the remodeling stage occurs 10+ days post-injury and is characterized by either functional maturation of myofibrils (if satellite cells are activated during regeneration), or adipogenesis (if FAPs are activated during regeneration and differentiate into adipocytes). While we were unable to study these populations directly as they are dynamic and not yet well-defined in rat models, we inferred their activity based on tissue-specific outcomes at our selected timepoints. Our 4-day timepoint captures the regeneration stage of muscle healing and our 14-day timepoint captures the remodeling stage. At day 4 post-revision there are reduced inflammatory markers and reduced IL-6 and Notch signaling in response to plasma. We suggest that these changes contribute directly to the pro-myogenic activation of satellite cells and a shift in FAP activity toward myofibroblast differentiation over adipogenesis. This is supported by 1) the decrease in transcripts associated with the adipogenesis pathway at day 14 following plasma treatment, and 2) the decrease in inter-fibrotic fat deposits in the plasma-treated rats. Together, this suggests that plasma’s contribution to muscle healing is through early upregulation and resolution of inflammation, which drives the downregulation of adipogenic FAP activity. While both inflammation and FAP activity are required to activate satellite cell function, timing is critical as prolongation of these stimuli reduces myogenic-specific differentiation. Thus, it is likely that plasma-induced molecular changes speed up and/or reduce negative events to drive more productive healing and healthier muscle tissue by 14 days post-treatment. The outcomes we show are limited to histological rather than functional changes, as the changes in the muscle are unlikely to measurably decrease function in this model. To test the functional impact of these changes, it may be beneficial to use plasma in a more complicated surgery or in an aged model to determine if it could reduce scar tissue, and fibrosis, or functionally enhance long-term healing.

Importantly, plasma can stimulate cellular processes, promote tissue regeneration, and modulate inflammation non-pharmacologically. Each plasma device produces a unique cocktail of reactive products, and thus plasma devices can be used in combination to tailor a desired outcome.^[83]^ Plasma devices may also be used in combination with other non-pharmacological therapies, such as electrical stimulation devices, which have been shown to promote similar tissue-specific outcomes such as enhanced angiogenesis and wound contraction.^[84]^ In fact, plasma and electrical stimulation devices have been used in combination in previous studies and have shown synergy in promoting cancer cytotoxicity.^[85]^ Finally, because plasma is not a drug-based therapy, it could be integrated into the clinical standard of post-operative care. Plasma has shown synergistic effects with antibiotics, and because the treatment times for plasma are relatively short, it could easily be integrated into operating room protocols to promote healing and prevent infection.^[86]^

Our study has limitations. The design of the in vivo study does not show a direct link between the reactive products produced by plasma and the effects that are observed in the treated tissue. However, we show that the plasma device does produce reactive species including ROS and RNS, and while the published mechanism of action for the device is considered the local and efficient transfer of heat, our method of moving the plume around the treated site greatly reduces the heat exposure of any single area of treated tissue.^[26]^ Additionally, the device itself only shows heat effects on tissue up to 0.2 mm deep, which is a highly limited area even without movement of the plume.^[87]^ Further, the catalase protein and transcriptional changes observed 6 h after treatment, and the long-term changes to tissue and signaling pathways are not characteristic of heat exposure, but of reactive species exposure.^[88,89]^ Despite this, we acknowledge that upregulation of catalase is a downstream effect of ROS/RNS pathway signaling and does not represent a direct readout of the proportion of these species that are present in the treated incision site. A second limitation of the study is the inherent variability present in the model. While using an outbred strain of rats is beneficial to explore how plasma treatment may have variable effects in a diverse population, it presents a challenge when drawing conclusions about the effect of plasma treatment on tissue. Further, rat and human skins differ in their healing processes. Rats tend to heal through a process of wound contraction whereas humans heal through a process of re-epithelialization.^[90]^ Thus, the effect of plasma treatment on connective tissue, collagen structures, and muscle fibers in rats does not directly translate to human biology. Finally, the timepoints chosen provide only a small snapshot of a highly complex process. Longer timepoints of 20+ days post-surgery would be needed to draw more definitive conclusions about functional muscle repair following plasma treatment. Thus, we did our best to interpret the results, but it should be noted that the overall effects of reactive species on signaling and satellite cell differentiation in muscle repair can be highly variable and remain controversial.

Our study reveals that Renuvion plasma treatment of a surgical incision enhances muscle regeneration through immediate changes to the treated tissue in a localized, tissue-specific manner. Plasma also stimulates an immune response that surpasses the normal immune response to tissue injury. Antioxidant and damage repair pathways are upregulated at 6 h in response to plasma treatment with a corresponding decrease in apoptosis pathway activity. Ultimately these early changes to the plasma-treated tissue result in reduced inter-fibrotic fat and adipogenesis during the later stages of healing. Our results provide new insights into the immediate and long-term effects of plasma treatment of muscle tissue within a surgical incision and provide merit to further investigate the clinical use of cold plasma as an accompanying therapeutic in surgical procedures.

## 4. Experimental Section

### Renuvion Device:

All experiments were conducted using the Renuvion device (Apyx Medical Corporation, Clearwater, FL). Plasma was generated using the Bovie Ultimate Electrosurgical Generator (Ref: BVX-200H). Animals were treated with the Apyx 44 Derm Handpiece (Ref: APYX-044-DERM). All treatments used the settings: 28 W, and 4 L min^−1^ helium flow rate. These settings were based on the safety and efficacy documentation used for FDA approval of the device and the settings used by clinicians for dermal and subdermal treatments.^[91]^

### Renuvion pH and Chemical Product Analysis:

MilliQ H2O (1 mL, 3 replicates) was treated with Renuvion at 28 W, and 4 L min^−1^ helium flow rate at ≈2 mm from the water surface at timepoints of 0, 5, 10, 20, 30, and 60 s in a flat-bottom 24-well plate (Thermo Fisher Scientific Inc., Waltham, MA USA). The applicator was moved across the water surface during each treatment session to ensure maximum exposure. The treated water was probed using a pH meter (Thermo Fisher Scientific Inc. Waltham, MA USA). For chemical product analysis, the treated water was prepared in a 96-well plate for the quantification of species. For the detection of hydrogen peroxide, 100 μL of 1 m potassium iodide and 50 μL of 10 mm phosphate buffer at a pH of 7 was added to 50 μL of plasma-treated water, incubated for 30 min in the dark, and measured at 390 nm using a plate reader (Tecan Infinite M1000 spectrometer). For the detection of nitrite species, 50 μL of Griess reagent (Thermo Fisher Scientific Inc., Waltham, MA USA) was added to 50 μL of plasma-treated water, incubated in the dark for 30 min, and measured at 548 nm on a plate reader (Tecan Infinite M1000 spectrometer). For the detection of nitrate species, the Nitrate Spectraquant Assay (Sigma-Aldrich, Dorset UK) was used. 10 μL of plasma-treated water was added to 80 μL of NO3^−1^, and 10 μL of NO3^−2^, incubated in the dark for 10 min, and measured at 548 nm on a plate reader. Values were converted to μm using a standard concentration curve.

### Orthopedic Animal Model and Plasma Treatment: 6 h Post-Plasma Treatment Rats:

A total of 12 (*n* = 6 untreated and *n* = 6 plasma-treated) 10–11-week-old male Sprague-Dawley rats (360–506 g) were used for this study. All procedures were approved by the University of Pennsylvania IACUC protocol #806810. Animals were anesthetized via cage induction using Isoflurane in an O_2_ carrier. Perioperative medications administered included a single dose of meloxicam (2 mg kg^−1^ subcutaneously) (Loxicom, Biloxi, MS), buprenorphine extended-release injectable suspension (0.65 mg kg^−1^ bwt subcutaneously) (Fidelis Animal Health, Inc, North Brunswick, NJ) and subcutaneous Lactated Ringers solution (10 mL kg^−1^) (Medex Medical Supply, Passaic, NJ). The femur was accessed via a lateral approach through the anatomical tissue plane between the vastus lateralis and the biceps femoris. A combination of blunt and sharp dissection was used to expose the lateral surface of the femur. Renuvion plasma was applied to all muscle areas of the incision until slightly discolored. The distance between the plasma device and the tissue was between 6–10 mm from the surface of the incision, and the incision treatment time was ≈30 s (or until all exposed tissue was contacted by the plasma). The incisions of all animals, regardless of treatment, were lavaged copiously with 20 mL sterile saline, and the discolored tissue was gently debrided from plasma-treated animals. The muscle was closed in a simple continuous pattern using 4/0 polyglactin 910 and the skin was closed in a simple continuous pattern using 4/0 polyglecaprone. The animals were sacrificed 6 h after surgery via intracardiac injection of a pentobarbital-based euthanasia solution under general anesthesia (Vortech Pharmaceuticals Ltd, Dearborn, MI). The vastus lateralis was excised and processed for histology and the biceps femoris was excised and processed for RNA extraction and protein isolation. Day 4 and 14 post-plasma treatment rats: A total of 12 (*n* = 6 untreated and *n* = 6 plasma-treated) 10–11-week-old male Sprague-Dawley rats were used for each day 4 and day 14 groups. For index surgery, anesthetic, and medication protocols were identical to those previously described except for animals receiving 3 doses of meloxicam 24 h apart. The femur was accessed through the same surgical plane as described above. A combination of blunt and sharp dissection was used to expose the lateral surface of the femur. A 0.8 mm screw (RISystem AG, Switzerland) was placed at the proximal and distal-most extents of the exposed femur. A unicortical open drill hole was created in the center of the lateral aspect of the femur to mimic a fracture. A collagen sponge (Davol Inc., Warwick, RI) was then placed over the screw heads and saturated with 250 uL of sterile saline. The muscle was closed in a simple continuous pattern using 4/0 polyglactin 910 and the skin was closed in a simple continuous pattern using 4/0 polyglecaprone. A subcutaneous peri-incisional infiltration was performed with liposome injectable suspension (Elanco, Greenfield, IN). One week later the animals were anesthetized and administered the same medications as previously described. An identical approach to the lateral femur was made and the screws were removed. Renuvion plasma was applied to all muscle areas of the incision as described above. The incision was lavaged copiously and the discolored tissue was gently debrided. The incision was lavaged with 20 mL of sterile saline (in treated animals) or 3% iodine solution (in untreated animals) with a contact time of 5 min. An 8-hole polyetheretherketone (PEEK) plate (RISystem AG, Switzerland) was then placed on the lateral aspect of the femur and secured with 4 screws to avoid the previously made holes in the cortex. The muscle was closed in a simple continuous pattern using 4/0 polyglactin 910 and the skin was closed in a simple continuous pattern using 4/0 polyglecaprone. A subcutaneous peri-incisional infiltration was performed with bupivacaine liposome injectable suspension (Elanco, Greenfield, IN). The animals were humanely euthanized either 4 or 14 days after surgery via intracardiac injection of a pentobarbital-based euthanasia solution under general anesthesia (Vortech Pharmaceuticals Ltd, Dearborn, MI).

### Histology and Immunohistochemistry: Tissue Preparation:

Excised tissue was fixed in 4% paraformaldehyde (PFA) for up to 48 h and then transferred to 70% ethanol for 24 h. The tissue was then progressively dehydrated in graded alcohols and xylenes before being embedded in paraffin wax blocks (Thermo Fisher Scientific Inc., Waltham, MA USA). The orientation of the sample when embedding in paraffin was bisected perpendicular to and in the center of each incision. SEM Imaging: The incision site or treated site was excised from the larger muscle sample and fixed in 4% paraformaldehyde for at least 24 h. After fixation, the muscle was serially dehydrated through a graded alcohol series from 70 to 100% with each phase lasting at least 6 h. After dehydration, the samples were dried for 72 h. The samples were then sputter-coated with gold and imaged with a Tabletop Hitachi Scanning Electron Microscope. Masson’s Trichrome Staining: Masson’s Trichrome was used to visualize muscle fiber and connective tissue structure (Millipore, Burlington, MA USA). Toluidine Blue Staining: Toluidine Blue staining (Toluidine Blue O, Thermo Fisher Scientific Inc., Waltham, MA, USA, 1.1 mg mL^−1^ in 1% NaCl, 8% ethanol solution) was used to visualize mast cells (dark purple) between the muscle fibers and connective tissue (light blue). Slides were imaged at 20× with 5 images being taken of different non-overlapping regions of the fibrotic tissue for each animal. Mast cell counts/area were calculated for each of the technical replicates and averaged to report the number of mast cells present in the fibrotic tissue in the treated site of each animal. Immunohistochemistry: Immunohistochemistry (IHC) DAB kit (Vector, Newark, CA USA) and an antibody against myeloperoxidase (Abcam, Waltham, MA USA) were used to visualize neutrophils in the fibrotic tissue. Slides were imaged at 20× with 5 images being taken of different non-overlapping regions of the fibrotic tissue in the treated site of each animal. MPO+ cell counts/area were calculated for each of the technical replicates and averaged to report the number of neutrophils present in the fibrotic tissue of each animal. Picrosirius Red Staining: Picrosirius red staining (Thermo Fisher Scientific Inc., Waltham, MA USA) was used to visualize collagen fibers (red) and muscle tissue (orange/yellow). 3–10 nonoverlapping images were taken at 20× of each incision site. Fat area per total fibrotic area was calculated for each image and averaged to report the total percent fat area within the fibrotic region of each animal.

### Protein Extraction and Quantification:

Protein was isolated from excised muscle tissue using tissue protein extraction reagent (TPER) (Thermo Fisher Scientific Inc., Waltham, MA USA) and M tubes (Miltenyi Biotec, Gaithersburg, MD) to homogenize the tissue using a gentleMACS tissue dissociator (Miltenyi Biotec, Gaithersburg, MD). The supernatant was collected from the homogenate after it was spun down at 1000 g for 5 min, followed by another centrifuge step at 10 000 g for 5 min. Protein concentration was quantified using the BCA assay (Thermo Fisher Scientific Inc., Waltham, MA USA). Protein was separated using sodium dodecyl sulfate-polyacrylamide gel electrophoresis (SDS-PAGE) and transferred onto a membrane (BioRad, Philadelphia, PA). The proteins were detected using the following rat anti-rabbit primary antibodies: anti-catalase (1:1000) (Abclonal, Woburn, MA), anti-myeloperoxidase (1:1000) (Abcam, Waltham, MA), and anti-GAPDH (1:20 000) (Abclonal, Woburn, MA). The protein was visualized using a chemiluminescence detector and developer (Azure Biosystems, Dublin, CA). The bands were analyzed in ImageJ.

### RNA Isolation, Library Construction, and Sequencing Analysis: RNA Isolation:

The muscle tissue was homogenized by resuspending crushed muscle in TRIzol reagent (Invitrogen, Waltham, MA USA) at 1 mL of reagent per 0.1 g of muscle. Then the tissue was dissociated by using M tubes and a gentle MACS tissue dissociator (Miltenyi Biotec, Gaithersburg, MD USA). The RNA was extracted using the Macherey-Nagel NucleoSpin RNA Midi Kit (Macherey-Nagel, Allentown, PA USA) after chloroform phase separation. RNA Sequencing: RNA samples were sent to an off-site laboratory (Novogene, Sacramento, CA USA) for validation of RNA integrity and sequencing using standard procedures for library construction, amplification, and sequencing. For the analysis, the sequencing data was converted to raw read counts from gene expression values and mapped to the reference genome Rattus norvegicus by Novogene. Outlier and DEG Analysis: Differential gene expression, gene ontology, and robust principal component analysis were done in R Statistical Software (v.4.3.3; R Core Team 2024) using the DESeq2, clusterProfiler, and rrcov packages.^[92–94]^ The transcripts of ≈32 500 genes were measured. First, the raw counts were normalized, and the variance of the samples was visualized using robust principal component analysis. The identified outlier samples were discarded (*n* = 3 per timepoint) and the remaining samples (*n* = 9 per timepoint) were analyzed for differentially expressed genes (DEGs). The significant DEGs presented an adjusted *p*-value < 0.05 using the Benjamini-Hochberg-adjusted value. Gene Set Enrichment Analysis: Gene set enrichment analysis (GSEA) was done using the GSEA software from the Molecular Signature Database (MSigDB).^[95]^ The input gene list for GSEA was extracted from the DESeq2 analysis. Gene transcripts with an average expression value <10 across all nine samples/timepoint were removed prior to running GSEA. GSEA was run with the phenotype permutation and a minimum gene list set to 10 for clustering. The genesets used were the molecular signatures databases curated and Hallmark and the mitocarta 3.0 database.^[96]^

### Cytokine Array:

Protein was isolated as described above. Protein samples were sent to an off-site laboratory (RayBiotech, Peachtree Corners, GA) and quantitative proteomics were performed using a Multiplex ELISA panel of rat-specific cytokines. Expression data for each treatment group (untreated and plasma-treated) and timepoint (day 4 and day 14) were averaged, and a student’s t-test was used to determine the significance of the difference in expression.

### Statistical Analysis:

For Figure 1A, *n* = 3 for all timepoints. For Figures 2A and 3D, the untreated group had *n* = 4 and the treated group had *n* = 5. A Mann–Whitney test was used to determine the significance between the groups. For Figure 2B,C the untreated group had *n* = 4 and the treated group had *n* = 5. A student’s t-test was used to determine the significance between the groups. For Figures 3 and 4, the RNA sequencing data was analyzed using a robust PCA using R Statistical Software (v.4.3.3; R Core Team 2024) using the DESeq2, clusterProfiler, and rrcov packages to exclude outliers. After excluding outliers, the RNA sequencing data included *n* = 4 for the untreated group and *n* = 5 for the treated group. For Figure 3D a Man Whitney test was used to determine significance between groups. For Table 1, a student’s t-test was used to determine the significance between groups. Day 4 untreated *n* = 5, Day 4 treated *n* = 3, Day 14 untreated *n* = 5, Day 14 treated *n* = 4. For Figure 5B,C *n* = 4 for both groups, and a student’s t-test was used to determine statistical significance. *p* < 0.05 indicates statistical significance. GraphPad Prism 10.4.1 was used for all analyses.

## Supplementary Material

Supplementary Material

Supporting Information is available from the Wiley Online Library or from the author.

## Figures and Tables

**Table 1.** Cytokine expression levels (pg mL^−1^) determined by ELISA array in the homogenized muscle of untreated and plasma-treated animals 4- and 14-days post-revision surgery. The table includes only cytokines that were significantly changed (*p* < 0.05) with plasma treatment. n≥3 at all timepoints and treatments. P-values determined by student’s t-test. Expression Levels of Cytokines which were Significantly Lowered with Plasma Treatment.

Average Cytokine Expression [pg mL^−1^] ± SEM						
Cytokine	Day 4 Untreated	Day 4 Plasma	*p*-Value^a)^	Day 14 Untreated	Day 14 Plasma	*p*-Value^a)^
IL-4	0.91 ± 0.21	0.11 ± 0.10	0.015	0.31 ± 0.12	0.30 ± 0.18	0.943
CINC2	16.79 ± 2.76	15.67 ± 5.40	0.866	27.74 ± 3.04	10.50 ± 5.63	0.046
IL-13	5.66 ± 3.49	9.33 ± 0.90	0.361	17.36 ± 1.23	4.58 ± 1.96	0.002
L-Selectin	20.62 ± 8.34	13.71 ± 4.30	0.492	29.97 ± 6.02	4.58 ± 2.00	0.011
RAGE	91.89 ± 47.40	61.76 ± 32.60	0.619	53.55 ± 9.15	12.79 ± 6.59	0.009

a) *p*-Value determined by student’s t-test.

## Data Availability

The data that support the findings of this study are available in the supplementary material of this article.

## References

[1] Junker JPE, Kamel RA, Caterson EJ, Eriksson E, Adv. Wound Care 2013, 2, 348. 10.1089/wound.2012.0412 PMC3842869 24587972

[2] Bernhardt T, Semmler ML, Schäfer M, Bekeschus S, Emmert S, Boeckmann L, Oxid Med Cell Longev 2019, 3873928. 31565150 10.1155/2019/3873928 PMC6745145

[3] Ruff PG, Sterodimas A, Aesthetic Plast Surg 2024, 48, 612. 38097690 10.1007/s00266-023-03749-6 PMC10954941

[4] Laroussi M, Cold Gas Plasma Sources and the Science behind their Applications in Biology and Medicine.

[5] Lu X, Naidis GV, Laroussi M, Reuter S, Graves DB, Ostrikov K, Phys Rep 2016, 630, 1.

[6] Braný D, Dvorská D, Halašová E, Škovierová H, Int J Mol Sci 2020, 21, 8. 10.3390/ijms21082932 PMC7215620 32331263

[7] Elmore L, Minissale NJ, Israel L, Katz Z, Safran J, Barba A, Austin L, Schaer TP, Freeman TA, Biomedicines 2024, 12, 277. 38397879 10.3390/biomedicines12020277 PMC10886613

[8] Lou BS, Hsieh JH, Chen CM, Hou CW, Wu H, Chou PY, Lai C, Lee JW, Front. Bioeng. Biotechnol 2020, 8, 683. 32695763 10.3389/fbioe.2020.00683 PMC7338308

[9] Chernets N, Kurpad DS, Alexeev V, Rodrigues DB, Freeman TA, Plasma Processes Polym 2015, 12, 1400. 10.1002/ppap.201500140 PMC5667549 29104522

[10] Gupta KK, Routray W, Food Chem 2025, 472, 142960. 39842194 10.1016/j.foodchem.2025.142960

[11] Lin A, Truong B, Patel S, Kaushik N, Choi E, Fridman G, Fridman A, Miller V, Int. J. Mol. Sci 2017, 18, 966. 28467380 10.3390/ijms18050966 PMC5454879

[12] Thompson TP, Connelly A, Kelly S, Duncan RM, Maybin JA, McDonnell C, Melvage A, McClenaghan LA, Dedeloudi A, Lamprou DA, Schaer TP, Bourke P, Hickok NJ, Gilmore BF, Freeman TA, Adv. Ther 2025, 2400339. 10.1002/adtp.202400339 PMC12439852 40964152

[13] Ahn HJ, Il KK, Kim G, Moon E, Yang SS, Lee JS, PLoS One 2011, 6, 11. 10.1371/journal.pone.0028154 PMC3226649 22140530

[14] Arndt S, Unger P, Berneburg M, Bosserhoff AK, Karrer S, J. Dermatol. Sci 2018, 89, 28154181. 10.1016/j.jdermsci.2017.11.008 29191392

[15] Bolgeo T, Maconi A, Gardalini M, Gatti D, Di Matteo R, Lapidari M, Savioli Y, Piccioni A, Zanza C, J Pers Med 2023, 13, 5. 10.3390/jpm13050736 PMC10219374 37240907

[16] Abu Rached N, Kley S, Storck M, Meyer T, Stücker M, J. Clin. Med 2023, 12, 15. 10.3390/jcm12155121 PMC10419810 37568525

[17] Landscheidt K, Engelhardt C, Hernekamp JF, Goertz O, Adv. Skin Wound Care 2022, 35, 22486. 10.1097/01.ASW.0000891084.22486.a7 36409191

[18] Gonzales LISA, Qiao JW, Buffier AW, Rogers LJ, Suchowerska N, McKenzie DR, Kwan A, Biophys. Rev 2023, 4, 1. 10.1063/5.0089831 PMC10903421 38510160

[19] Hirst AM, Simms MS, Mann VM, Maitland NJ, O’Connell D, Frame FM, Br. J. Cancer 2015, 112, 11536. 10.1038/bjc.2015.113 PMC4454887 25839988

[20] Li X, Li M, Ji N, Jin P, Zhang J, Zheng Y, Zhang X, Li F, LWT 2019, 115, 108447.

[21] Schmidt A, Dietrich S, Steuer A, Weltmann KD, von Woedtke T, Masur K, Wende K, J. Biol. Chem 2015, 290, 6731. 25589789 10.1074/jbc.M114.603555 PMC4358097

[22] Hirasawa I, Odagiri H, Park G, Sanghavi R, Oshita T, Togi A, Yoshikawa K, Mizutani K, Takeuchi Y, Kobayashi H, Katagiri S, Iwata T, Aoki A, PLoS One 2023, 18, 0292267. 10.1371/journal.pone.0292267 PMC10584116 37851686

[23] Hua D, Cai D, Ning M, Yu L, Zhang Z, Han P, Dai X, J. Cancer 2021, 12, 5977. 34476012 10.7150/jca.54528 PMC8408125

[24] Bekeschus S, Schmidt A, Weltmann KD, von Woedtke T, Clin. Plasma Med 2016, 4, 19.

[25] Bekeschus S, von Woedtke T, Emmert S, Schmidt A, Redox Biol 2021, 46, 102116. 34474394 10.1016/j.redox.2021.102116 PMC8408623

[26] Ruff PG, Doolabh V, Zimmerman EM, Gentile RA, Dermatolog. Rev 2020, 1, 108.

[27] Gentile RD, McCoy JD, Facial Plast. Surg. Clin. North Am 2020, 28, 75. 31779944 10.1016/j.fsc.2019.09.007

[28] Filis K, Galyfos G, Sigala F, Zografos G, J. Vasc. Surg. Cases Innov. Tech 2020, 6, 152. 32154472 10.1016/j.jvscit.2020.01.008 PMC7056604

[29] Gentile RD, Facial Plast. Surg. Aesthet. Med 2020, 22, 304. 32379988 10.1089/fpsam.2020.0070 PMC7374633

[30] Moncada S, Nichols C, Aesthet. Surg. J. Open Forum 2022, 4, ojac041. 10.1093/asjof/ojac041 PMC9336572 35912360

[31] Holcomb JD, Schucker A, Lasers Surg. Med 2020, 52, 23. 31587330 10.1002/lsm.23167 PMC7004100

[32] Duncan DI, Combining Helium Plasma-Driven Radiofrequency with Nanofat for Contouring [Internet]. Available from, www.intechopen.com (accessed: March 2025).

[33] Carosio S, Berardinelli MG, Aucello M, Musarò A, Ageing Res. Rev 2011, 10, 35. 19683075 10.1016/j.arr.2009.08.001

[34] ten Broek RPG, Issa Y, van Santbrink EJP, Bouvy ND, Kruitwagen RFPM, Jeekel J, Bakkum EA, Rovers MM, van Goor H, BMJ 2013, 34, f5588-. 10.1136/bmj.f5588 PMC3789584 24092941

[35] Brunelli S, Roverequerini P, Pharmacol Res 2008, 58, 117. 18639637 10.1016/j.phrs.2008.06.008

[36] Dort J, Fabre P, Molina T, Dumont NA, Stem Cells Int 2019, 2019, 4761427. 31396285 10.1155/2019/4761427 PMC6664695

[37] Murphy MM, Lawson JA, Mathew SJ, Hutcheson DA, Kardon G, Development 2011, 138, 3625. 21828091 10.1242/dev.064162 PMC3152921

[38] Uezumi A, ichiro FS, Yamamoto N, Takeda S, Tsuchida K, Nat. Cell Biol 2010, 12, 143. 20081842 10.1038/ncb2014

[39] Joe AWB, Yi L, Natarajan A, Le Grand F, So L, Wang J, Rudnicki MA, Rossi FMV, Nat. Cell Biol 2010, 12, 153. 20081841 10.1038/ncb2015 PMC4580288

[40] Bekeschus S, Winterbourn CC, Kolata J, Masur K, Hasse S, Bröker BM, Parker HA, J. Leukoc Biol 2016, 100, 791. 26992432 10.1189/jlb.3A0415-165RR

[41] Duchesne C, Frescaline N, Blaise O, Lataillade JJ, Banzet S, Dussurget O, Rousseau A, mSphere 2021, 6, 3. 10.1128/mSphere.00217-21 PMC8265637 34133202

[42] Ng MFY, Int. Wound J 2010, 7, 55. 20409251 10.1111/j.1742-481X.2009.00651.x PMC7951407

[43] Markus I, Constantini K, Hoffman JR, Bartolomei S, Gepner Y, Eur J Appl Physiol 2021, 121, 969. 33420603 10.1007/s00421-020-04566-4

[44] Musarò A, Adv. Biol 2014, 2014, 612471.

[45] Larouche JA, Fraczek PM, Kurpiers SJ, Yang BA, Davis C, Castor-Macias JA, Sabin K, Anderson S, Harrer J, Hall M, Brooks SV, Jang YC, Willett N, Shea LD, Aguilar CA, Proc. Natl. Acad. Sci. U.S.A 2022, 119, 2111445119. 10.1073/pnas.2111445119 PMC9169656 35377804

[46] Laumonier T, Menetrey J, J Exp Orthop 2016, 3, 1. 27447481 10.1186/s40634-016-0051-7 PMC4958098

[47] TIPTON KD, WOLFE RR, Acta Physiol. Scand 1998, 162, 377. 9578384 10.1046/j.1365-201X.1998.00306.x

[48] Zhang JF, Liu J, Wu JL, Li WF, Chen ZW, Yang LS, Onco Targets Ther 2019, 12, 4129. 31239701 10.2147/OTT.S201799 PMC6553995

[49] Bean C, Verma NK, Yamamoto DL, Chemello F, Cenni V, Filomena MC, Chen J, Bang ML, Lanfranchi G, Cell Death Dis 2014, 5, 1. 10.1038/cddis.2013.525 PMC4040671 24434510

[50] Liu Z, Vuohelainen V, Tarkka M, Tenhunen J, Lappalainen RS, Narkilahti S, Paavonen T, Oksala N, Wu Z, Mennander A, Scand. J. Clin. Lab Invest 2010, 70, 1002217. 10.3109/00365511003663655 20233036

[51] Wang K, Yaghi OK, Spallanzani RG, Chen X, Zemmour D, Lai N, Chiu IM, Benoist C, Mathis D, Proc. Natl. Acad. Sci. USA 2020, 117, 5402. 32102913 10.1073/pnas.1922559117 PMC7071852

[52] Petit CS, Roczniak-Ferguson A, Ferguson SM, J. Cell Biol 2013, 202, 1107. 24081491 10.1083/jcb.201307084 PMC3787382

[53] Liu EA, Schultz ML, Mochida C, Chung C, Paulson HL, Lieberman AP, JCI Insight 2020, 5, 20. 10.1172/jci.insight.136676 PMC7605537 32931479

[54] Ehrlich KC, Baribault C, Ehrlich M, Int. J. Mol. Sci 2020, 21. 33182325 10.3390/ijms21218394 PMC7672584

[55] Ferreira DMS, Cheng AJ, Agudelo LZ, Cervenka I, Chaillou T, Correia JC, Porsmyr-Palmertz M, Izadi M, Hansson A, Martínez-Redondo V, Valente-Silva P, Pettersson-Klein AT, Estall JL, Robinson MM, Nair KS, Lanner JT, Ruas JL, Skelet. Muscle 2019, 9, 1. 31666122 10.1186/s13395-019-0214-1 PMC6822430

[56] Liang S, Zhou Y, Chang Y, Li J, Zhang M, Gao P, Li Q, Yu H, Kawakami K, Ma J, Zhang R, Cell. Mol. Life Sci 2024, 81, 158. 38556571 10.1007/s00018-024-05189-0 PMC10982097

[57] Uhlén M, Fagerberg L, Hallström BM, Lindskog C, Oksvold P, Mardinoglu A, Sivertsson Å, Kampf C, Sjöstedt E, Asplund A, Olsson I, Edlund K, Lundberg E, Navani S, Szigyarto CA, Odeberg J, Djureinovic D, Takanen JO, Hober S, Alm T, Edqvist PH, Berling H, Tegel H, Mulder J, Rockberg J, Nilsson P, Schwenk JM, Hamsten M, von Feilitzen K, Forsberg M, , Science 2015, 347, 6220. 10.1126/science.1260419 25613900

[58] Stelzer G, Rosen N, Plaschkes I, Zimmerman S, Twik M, Fishilevich S, Stein TI, Nudel R, Lieder I, Mazor Y, Kaplan S, Dahary D, Warshawsky D, Guan-Golan Y, Kohn A, Rappaport N, Safran M, Lancet D, Curr. Protoc. Bioinformatics 2016, 54, 1. 10.1002/cpbi.5 27322403

[59] Kinney CJ, Endocr. Regul 2021, 55, 89. 34020530 10.2478/enr-2021-0011 PMC9202446

[60] Moffat C, Bhatia L, Nguyen T, Lynch P, Wang M, Wang D, Ilkayeva OR, Han X, Hirschey MD, Claypool SM, Seifert EL, J. Lipid Res 2014, 55, 2458. 25114170 10.1194/jlr.M046961 PMC4242439

[61] Cai LQ, Li XC, Wang YY, Chen YX, Zhu XY, Zuo ZY, , Inflammation 2024, 47, 6. 10.1007/s10753-024-02032-3 PMC11607023 38653921

[62] Doi R, Endo M, Yamakoshi K, Yamanashi Y, Nishita M, Fukada S, Minami Y, Genes Cells 2014, 19, 287. 24475942 10.1111/gtc.12132

[63] Cai M, Shao J, Yung B, Wang Y, Gao NN, Xu X, , Neural Regen. Res 2022, 17, 845. 34472485 10.4103/1673-5374.322474 PMC8530132

[64] Postler A, Lützner C, Beyer F, Tille E, Lützner J, BMC Musculoskelet Disord. 2018, 19, 55. 29444666 10.1186/s12891-018-1977-y PMC5813428

[65] Bari TJ, Karstensen S, Sørensen MD, Gehrchen M, Street J, Dahl B, Spine Deform 2020, 8, 1341. 32607936 10.1007/s43390-020-00164-8

[66] Heckmann ND, Yang J, Ong KL, Lau EC, Fuller BC, Bohl DD, Della Valle CJ, J. Arthroplasty 2021, 36, 1779.e2. 33504458 10.1016/j.arth.2020.12.035

[67] Becker M, Joseph SS, Garcia-Carrizo F, Tom RZ, Opaleva D, Serr I, Tschöp MH, Schulz TJ, Hofmann SM, Daniel C, Cell Metab 2023, 35, 1736. 37734370 10.1016/j.cmet.2023.08.010 PMC10563138

[68] Mourikis P, Sambasivan R, Castel D, Rocheteau P, Bizzarro V, Tajbakhsh S, Stem Cells 2012, 30, 243. 22069237 10.1002/stem.775

[69] Bhattacharjee A, Shukla M, Yakubenko VP, Mulya A, Kundu S, Cathcart MK, Free Radic. Biol. Med 2013, 54, 23124025. 10.1016/j.freeradbiomed.2012.10.553 PMC3534796 23124025

[70] Hardee JP, Martins KJB, Miotto PM, Ryall JG, Gehrig SM, Reljic B, Naim T, Chung JD, Trieu J, Swiderski K, Philp AM, Philp A, Watt MJ, Stroud DA, Koopman R, Steinberg GR, Lynch GS, Mol. Metab 2021, 45, 101157. 33359740 10.1016/j.molmet.2020.101157 PMC7811171

[71] Lozano DD, Arch. Surgery 1999, 134, 1079. 10.1001/archsurg.134.10.1079 10522850

[72] Riuzzi F, Sorci G, Sagheddu R, Chiappalupi S, Salvadori L, Donato R, J. Cachexia Sarcopenia Muscle 2018, 9, 1213. 30334619 10.1002/jcsm.12350 PMC6351676

[73] Htet Hlaing K, Clément MV, Free Radic. Res 2014, 48, 996. 25005256 10.3109/10715762.2014.942842

[74] Su LJ, Zhang JH, Gomez H, Murugan R, Hong X, Xu D, , Oxid. Med. Cell Longev 2019, 2019, 5080843. 31737171 10.1155/2019/5080843 PMC6815535

[75] Wang B, Laskin A, J. Geophys. Res., Atmospheres 2014, 119, 3335.

[76] Dong Y, Wang Z, Front. Bioeng. Biotechnol 2023, 11, 1304835. 38149175 10.3389/fbioe.2023.1304835 PMC10749972

[77] Zahid AA, Ahmed R, ur Rehman SR, Augustine R, Hasan A, in 2019 41st Annual International Conference of the IEEE Engineering in Medicine and Biology Society (EMBC), IEEE, 2019, p. 3939. 10.1109/EMBC.2019.8856469 31946734

[78] Martin P, Leibovich SJ, Trends Cell Biol 2005, 15, 599. 16202600 10.1016/j.tcb.2005.09.002

[79] Musarò A, Adv. Biol 2014, 2014, 612471.

[80] Hoffman DB, CJ RP, Sorensen JR, Corona BT, Greising SM, Connect. Tissue Res 2022, 63, 124. 33535825 10.1080/03008207.2021.1886285 PMC8364566

[81] Hempel N, Trebak M, Cell Calcium 2017, 63, 70. 28143649 10.1016/j.ceca.2017.01.007 PMC5466514

[82] Theret M, Rossi FMV, Contreras O, Front Physiol 2021, 12, 673404. 33959042 10.3389/fphys.2021.673404 PMC8093402

[83] Madigan CD, O’Sullivan D, O’Neill L, Kavanagh DF, Clin. Plasma Med 2020, 19–20, 100108.

[84] Preetam S, Ghosh A, Mishra R, Pandey A, Roy DS, Rustagi S, Malik S, RSC Adv 2024, 14, 32142. 39399261 10.1039/d4ra04258a PMC11467653

[85] Wolff CM, Kolb JF, Weltmann KD, von Woedtke T, Bekeschus S, Cancers (Basel) 2020, 12, 845. 32244543 10.3390/cancers12040845 PMC7226014

[86] Maybin JA, Thompson TP, Flynn PB, Skvortsov T, Hickok NJ, Freeman TA, Gilmore BF, Biofilm 2023, 5, 100122. 37214348 10.1016/j.bioflm.2023.100122 PMC10196807

[87] Gentile RD, Facial Plast. Surg. Aesthet. Med 2020, 22, 304. 32379988 10.1089/fpsam.2020.0070 PMC7374633

[88] Sebok J, Édel Z, Váncsa S, Farkas N, Kiss S, Eross B, Török Z, Balogh G, Balogi Z, Nagy R, Hooper PL, Geiger PC, Wittmann I, Vigh L, Dembrovszky F, Hegyi P, Int. J. Hyperthermia 2021, 38, 1650. 34808071 10.1080/02656736.2021.2003445

[89] Powers SK, Ji LL, Kavazis AN, Jackson MJ, in Comprehensive Physiology, Wiley, Bota Rocan, FL, 2011, p. 941. 10.1002/cphy.c100054 PMC3893116 23737208

[90] Weber B, Lackner I, Haffner-Luntzer M, Palmer A, Pressmar J, Scharffetter-Kochanek K, Knöll B, Schrezenemeier H, Relja B, Kalbitz M, J. Transl. Med 2019, 17, 305. 31488164 10.1186/s12967-019-2052-7 PMC6728963

[91] Ruff PG, Doolabh V, Zimmerman EM, Gentile RA, Dermatolog. Rev 2020, 1, 108.

[92] Love MI, Huber W, Anders S, Genome Biol 2014, 15, 550. 25516281 10.1186/s13059-014-0550-8 PMC4302049

[93] Yu G, Wang LG, Han Y, He QY, OMICS 2012, 16, 284. 22455463 10.1089/omi.2011.0118 PMC3339379

[94] Filzmoser P, Todorov V, Inf. Sci. (N Y) 2013, 245, 4.

[95] Subramanian A, Tamayo P, Mootha VK, Mukherjee S, Ebert BL, Gillette MA, Paulovich A, Pomeroy SL, Golub TR, Lander ES, Mesirov JP, Proc. Natl. Acad. Sci. USA 2005, 102, 15545. 16199517 10.1073/pnas.0506580102 PMC1239896

[96] Rath S, Sharma R, Gupta R, Ast T, Chan C, Durham TJ, Goodman RP, Grabarek Z, Haas ME, Hung WHW, Joshi PR, Jourdain AA, Kim SH, Kotrys AV, Lam SS, McCoy JG, Meisel JD, Miranda M, Panda A, Patgiri A, Rogers R, Sadre S, Shah H, Skinner OS, To TL, Walker MA, Wang H, Ward PS, Wengrod J, Yuan CC, , Nucleic Acids Res 2021, 49, D1541. 33174596 10.1093/nar/gkaa1011 PMC7778944

