# Supplementary material for: Cold Plasma Generates a Localized Inflammatory Response and Promotes Muscle Repair
Source: Adv Ther (Weinh). Author manuscript; Available in PMC 2026 Jan 16. (PMC12807532; doi:10.1002/adtp.202500097)
Supplement: Supplementary Material [file NIHMS2130332-supplement-Supplementary_Material.docx]

Supporting Information

Promoting an Enhanced Tissue Repair Response with Cold Plasma Stimulation

Carly J. Smith, Amanda R. Watkins, Abigail A. Lucas, Arianna J. Moniodes, Conn Ritchie, Thomas P. Thompson, Thomas P. Schaer, Brendan F. Gilmore, Noreen J. Hickok, Theresa A. Freeman*

**
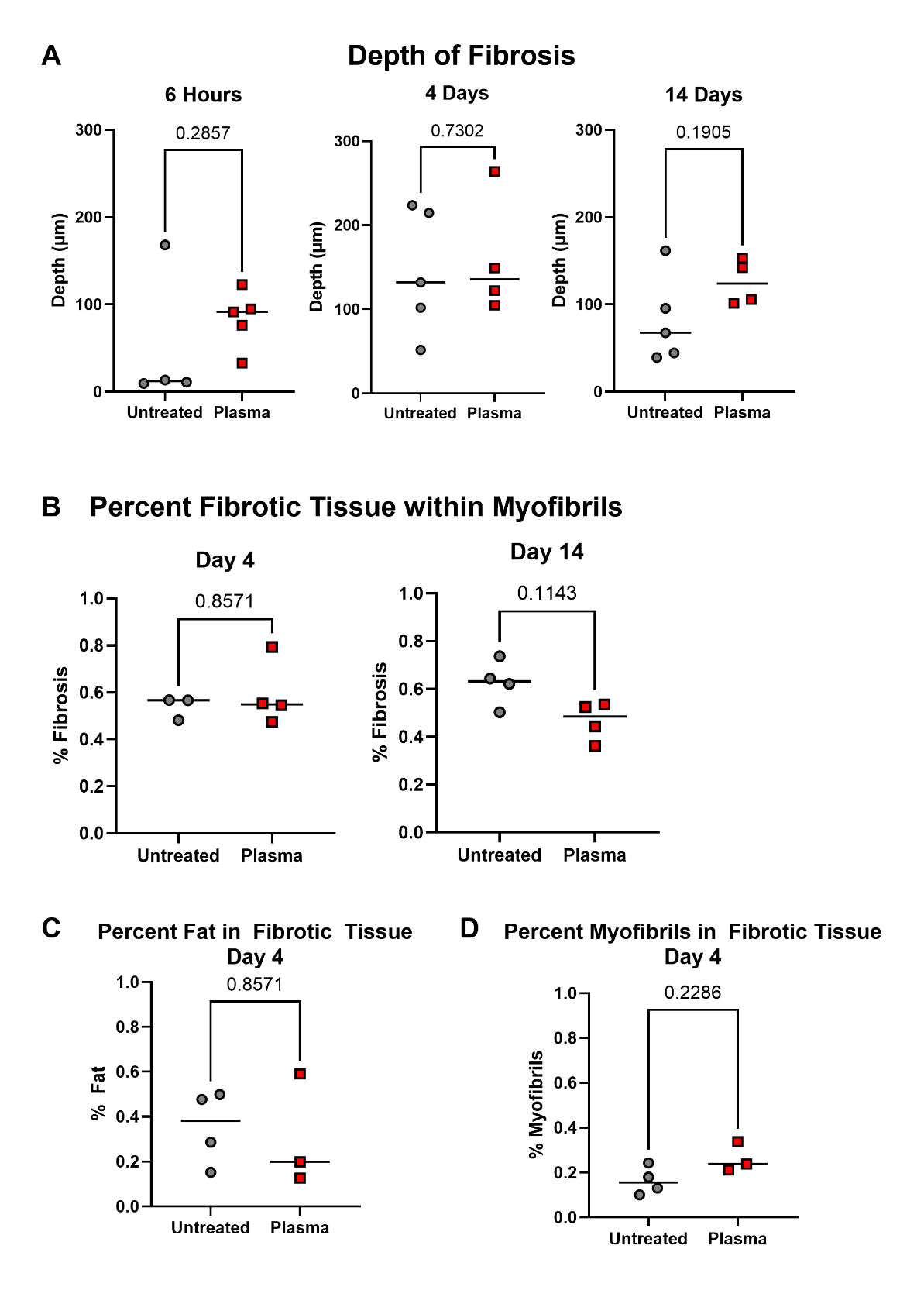
**

**Figure S1:** (A) Depth of fibrotic tissue measurements (µm) from the incision site of animals sacrificed 6 h, 4 days, and 14 days after index surgery (6 h cohort) or revision surgery (4- and 14-day cohorts). Measurements were taken as the width of the fibrotic tissue (visualized using Masson’s trichrome). Each datapoint represents the average measurements from 3 different sections taken from the mid-section of the operated vastus lateralis muscle. Significance determined using Mann-Whitney test (p<0.05). (B) Percent fibrotic tissue within the muscle fibers in animals sacrificed 4- or 14-days following revision surgery. Measurements were taken as the percent area of fibrotic tissue compared with the total area of the muscle tissue. Each datapoint represents the total percent fibrotic tissue in all muscle tissue from one section taken from the mid-section of the operated vastus lateralis muscle. Significance determined using Mann-Whitney test (p<0.05). (C) Percent fat in the fibrotic tissue in animals sacrificed 4 days following revision surgery. Each datapoint represents the total percent fat in all fibrotic tissue from one section taken from the mid-section of the operated vastus lateralis muscle. P-values determined using Mann Whitney test (p<0.05). (D) Percent myofibrils in the fibrotic tissue in animals sacrificed 4 days following revision surgery. Each datapoint represents the total percent myofibrils in all fibrotic tissue from one section taken from the mid-section of the operated vastus lateralis muscle. P-values determined using Mann Whitney test (p<0.05).

**
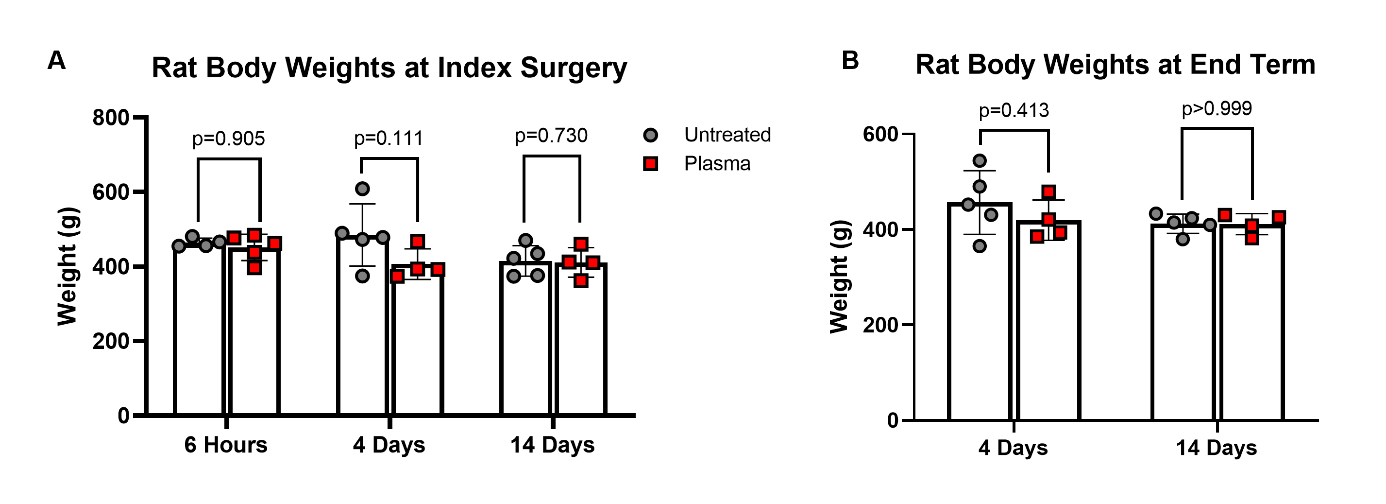
**

**Figure S2:** (A) Body weights (g) of 6 h, 4-day, and 14-day cohorts of rats at the time of index surgery. (B) Body weights (g) of 4-day, and 14-day cohorts of rats at the time of sacrifice. The 6 h cohort of rats was sacrificed 6 h after index weight measurement with no expected change in weight.
